# Superparamagnetic α-Fe_2_O_3_/Fe_3_O_4_ Heterogeneous Nanoparticles with Enhanced Biocompatibility

**DOI:** 10.3390/nano11040834

**Published:** 2021-03-24

**Authors:** You Li, Zhou Wang, Ruijiang Liu

**Affiliations:** 1 School of Pharmacy, Jiangsu University, Zhenjiang 212013, China; 2221915006@stmail.ujs.edu.cn; 2 College of Vanadium and Titanium, Panzhihua University, Panzhihua 617000, China; pzhwangzhou@163.com

**Keywords:** magnetic α-Fe_2_O_3_/Fe_3_O_4_ heterogeneous nanoparticles, cytotoxicity, human hepatocytes L-02, blood routine, histopathological section observation

## Abstract

A novel type of magnetic α-Fe_2_O_3_/Fe_3_O_4_ heterogeneous nanoparticles was prepared via a facile solution combustion process with ferric nitrate and urea as raw materials, and they were characterized by XRD, SEM, TEM, and VSM techniques. The effects of the calcination temperature, the calcination time, the ratio of ferric nitrate and urea, and the heating rate on the relative content of Fe_3_O_4_ in the heterogeneous nanoparticles were investigated. The toxicity of α-Fe_2_O_3_/Fe_3_O_4_ heterogeneous nanoparticles to human hepatocytes L-02, the blood routine, and the histopathological section observation of mice were explored. The results showed that the ratio of ferric nitrate and urea was a key factor to affect the relative content of Fe_3_O_4_ in the heterogeneous nanoparticles. The calcination temperature and the calcination time had similar influences, and the corresponding calcination temperature and the calcination time were selected according to their own needs. The CCK8 results initially revealed that α-Fe_2_O_3_/Fe_3_O_4_ heterogeneous nanoparticles had no effect on cell viability when the concentration of the heterogeneous nanoparticles was less than 100 ng/mL, which suggested their excellent biocompatibility. At the same time, the tail vein administration concentration of 0.9 mg/kg had good biological safety.

## 1. Introduction

Magnetic nanoparticles are of great interest to scientists and technologists due to their favorable magnetic and electrical properties, especially iron-oxide-based nanoparticles (MFe_2_O_4_, M = Fe, Mn, Co, Zn, or Ni, etc.). Although MFe_2_O_4_ nanoparticles have exhibited excellent characteristics in the biomedical field, their safe application in the human body is a major problem owing to the heavy metal elements presented in MFe_2_O_4_ [1], which also limits their applications in the biomedical field. So, as one of the typical nanomaterials, spinel iron oxide nanoparticles without heavy metal elements have been widely applied in many aspects of the biomedicine field due to their good biocompatibility and no toxicity or low toxicity in organisms [2,3], such as magnetic resonance imaging (MRI) [4], clinical diagnosis and the treatment of diseases [5,6], magnetic carriers for drug targeting [7], catalysis [8,9,10,11,12], immune assays [13], targeting photodynamic therapy [14,15,16], cell separation [17], DNA extraction [18], and so on. Therefore, the preparations and applications of iron oxide nanoparticles have attracted more and more researchers [19,20].

Iron oxide nanomaterials were mainly divided into three types, i.e., α-Fe_2_O_3_, γ-Fe_2_O_3_, and Fe_3_O_4_ [21]. In biomedical research and application processes, the saturation magnetization of magnetic α-Fe_2_O_3_ nanoparticles was small, so their magnetic targeting was low, which limited the applications of iron oxide nanoparticles in various fields [22,23,24]. Although the saturation magnetization of Fe_3_O_4_ nanoparticles was the largest in these three types, their magnetic targeting was enhanced; however, the agglomeration of magnetic Fe_3_O_4_ nanoparticles was also enhanced, which was not conducive to regulating the dispersion degree of magnetic Fe_3_O_4_ nanoparticles, thereby limiting their wide applications [25]. Therefore, the combined application of α-Fe_2_O_3_/Fe_3_O_4_ heterogeneous materials might provide a feasible solution for this problem, and it has been documented that FDA-approved iron oxide nanoparticles (Fe_2_O_3_/Fe_3_O_4_) could be redeployed for the treatment and control of COVID-19 infection using molecular docking technology [26]. In order to meet the application requirements of iron oxide nanoparticles in living organisms, the preparation of magnetic iron oxide heterogeneous nanoparticles with controllable size, shape, composition, and structure, as well as suitable saturation magnetization for better in vivo applications, has become a major trend of research.

In this project, we successfully prepared novel magnetic α-Fe_2_O_3_/Fe_3_O_4_ heterogeneous nanoparticles via a facile solution combustion process. The innovation point was that we explored the process conditions of each stage that affect its performance, and under the appropriate raw material ratio and calcination conditions, we prepared the ideal nanomaterials. At the same time, as the most important detoxification organ of the human body, the liver played a significant role in the recognition and transport of nanoparticles into the human body [27,28], so we selected the human normal hepatocytes L-02 as the research object to investigate the cytotoxicity of magnetic α-Fe_2_O_3_/Fe_3_O_4_ heterogeneous nanoparticles. The blood routine and histopathological section observation of mice injected magnetic α-Fe_2_O_3_/Fe_3_O_4_ heterogeneous nanoparticles through the tail vein were detected.

## 2. Materials and Methods

### 2.1. Preparation and Characteristics of Magnetic α-Fe_2_O_3_/Fe_3_O_4_ Heterogeneous Nanoparticles

Magnetic α-Fe_2_O_3_/Fe_3_O_4_ heterogeneous nanoparticles were prepared via a facile solution combustion process with ferric nitrate and urea as raw materials. Typically, 15.13 g Fe(NO_3_)_3_·9H_2_O and the urea were dissolved in 25 mL of distilled water. The quantity of urea was determined according to the ratio of ferric nitrate and urea (1:1, 1:2, 1:3, 1:4, 1:5, 1:6), and the mixture was magnetically stirred at room temperature until a homogeneous solution was formed. The solution was poured into a crucible and calcined in the programmed temperature control furnace at various temperatures (250, 300, 350, 400, 450, 500, 600 °C) for different times (0.5, 1, 1.5, 2, 2.5, 3, 4 h) with various heating rates (1, 3, 5, 7 °C·min^−1^). When the calcination was out, the crucible in the programmed temperature control furnace was cooled naturally, and the product was ground and collected to obtain magnetic α-Fe_2_O_3_/Fe_3_O_4_ heterogeneous nanoparticles.

The phase identification and composition analyses of magnetic α-Fe_2_O_3_/Fe_3_O_4_ heterogeneous nanoparticles were characterized by D/max 2500 PC X-ray diffraction (XRD, Rigaku, Tokyo, Japan) with Cu-Kα radiation. The morphologies were investigated with scanning electron microscopy (SEM, JSM-7800F, JEOL, Tokyo, Japan) and transmission electron microscopy (TEM, JEM-2100, JEOL, Tokyo, Japan). The magnetic properties of the nanomaterials were obtained by an ADE DMS-HF-4 vibrating sample magnetometer (VSM). The saturation magnetizations could be obtained from the measured hysteresis loops.

### 2.2. In Vitro Cytotoxicity Assay

Human L-02 hepatocytes (Shanghai Institute of Cell Science, Chinese Academy of Sciences, Shanghai, China) were cultured in RPMI 1640 medium (Gibco) supplemented with 10% fetal bovine serum (Gibco) at 37 °C in a humidified and 5% CO_2_ incubator. Cell lines were maintained with regular passaging, and the medium was replaced every 2 days. L-02 cells in 24-well plates were treated with different concentrations of α-Fe_2_O_3_/Fe_3_O_4_ heterogeneous nanoparticles for 24 h. Next, cells were fixed with 4% paraformaldehyde for 30 min and then stained with Perls stain staining solution at 37 °C for 30 min. After washing twice with deionized water, cells were counterstained with Perls for 1 min and then prepared for observation under a microscope (Olympus, Tokyo, Japan).

The Cell Counting Kit-8 (CCK-8) assay was performed according to the manufacturer’s instructions (Beyotime, Nantong, China). Cells were inoculated in a 96-well culture plate at a density of 4.0 × 10^3^/100 μL per well. The following day, the cells were grown in medium containing different concentrations of α-Fe_2_O_3_/Fe_3_O_4_ for 24 h. Before detection, 10 μL of CCK-8 was added to each well, and the plate was further incubated at 37 °C for 1 h. The absorbance of each well at 450 nm was read with a fully automatic microplate reader (SpectraMAX 190, Molecular Devices, California, USA).

After treating with different concentrations of α-Fe_2_O_3_/Fe_3_O_4_ nanomaterials for 24 h in L-02 cells, cell apoptosis was quantitatively determined with double stain with Annexin-V-FITC and Propidium Iodide (PI) following the manufacturer’s protocol (KeyGEN Biotech, Nanjing, China). Briefly, cells were digested with 0.25% trypsin without EDTA, washed by PBS, stained with Annexin-V-FITC and PI for 15 min in the dark, and then analyzed by flow cytometry (Calibur, BD Biosciences, New Jersey, USA). The number of apoptotic cells was analyzed using Flowjo V10 software and expressed as a percentage of the number of total cells. After the intervention of different concentrations of α-Fe_2_O_3_/Fe_3_O_4_ heterogeneous nanoparticles for 24 h, the supernatants were collected for detecting total antioxidant capacity (T-AOC) and malondialdehyde (MDA) concentration using ELISA kits (Beyotime, Nantong, China) according to the manufacturer’s instructions.

### 2.3. In Vivo Toxicity Assay

The local animal ethics committee approved all animal experiments (SYXK 2018-0053). To detect the effect of Fe_2_O_3_/Fe_3_O_4_ heterogeneous nanoparticles on the adult C57 mice, blood routine and blood biochemistry tests were performed. The 200 μL Fe_2_O_3_/Fe_3_O_4_ heterogeneous nanoparticle solutions with various concentrations of 0.9, 4.5, 45, and 450 mg/kg were separately injected via the tail vein, and the control group was injected with the same volume of normal saline. On Days 0, 14, and 28 after injection, the mice were fasted the night before each blood sampling time point to prevent some components in the food from interfering with the blood routine and blood biochemical detection. Blood samples from the orbital artery plexus were collected and divided into two parts. One part was dripped into the whole blood of the test tube added with anticoagulant heparin sodium for routine blood analysis. In another test tube with separation gel, serum was separated for blood biochemical detection. To ensure the quality of collection blood, the mice were prevented from excessive struggling and shocking during the process of blood collection.

Liver and kidney tissues were dissected and collected from mice that had been sacrificed by cervical dislocation under anesthesia. The tissues were fixed in 4% paraformaldehyde (PFA) for 12 h at 4 °C. Subsequently, they were dehydrated with ethanol and xylene, embedded in paraffin, and sliced continuously (5 μm in thickness). The tissue sections were incubated in a hematoxylin staining solution for 5 min and washed with flowing water for 20 min. After rinsing with water for 15–20 min and PBS for 30 s (nuclei turn blue), tissue sections were placed in eosin staining solution for 30 s; afterward, they were dehydrated in ethanol and xylene and then sealed with neutral resin.

All experiments were performed at least in triplicate. A comparison of different groups was determined using Student’s t-test. A value of *p* < 0.05 was considered statistically significant. The statistical data were analyzed by SPSS software.

## 3. Results and Discussion

### 3.1. Characteristics of Magnetic α-Fe_2_O_3_/Fe_3_O_4_ Heterogeneous Nanoparticles

**Figure 1:**
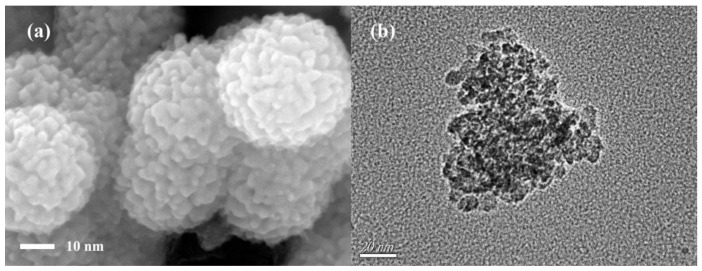
SEM morphology (**a**) and TEM image (**b**) of Fe_2_O_3_/Fe_3_O_4_ heterogeneous nanoparticles calcined at 350 °C for 2 h with the heating rate of 3 °C/min and the molar ratio of ferric nitrate and urea of 1:4.

The SEM morphology and TEM image of α-Fe_2_O_3_/Fe_3_O_4_ heterogeneous nanoparticles calcined at 350 °C for 2 h with the heating rate of 3 °C/min and the molar ratio of ferric nitrate and urea of 1:4 are revealed in Figure 1. From the SEM morphology (shown in Figure 1a), it can be seen that the size distribution of magnetic α-Fe_2_O_3_/Fe_3_O_4_ heterogeneous nanoparticles was uniform. The nanomaterials were composed of several nanograins, which resulted in irregular sphere particles. The roughness of the particle surface increased the specific surface area of the nanoparticles, and the average particle size of them was around 42 nm. The TEM image of magnetic α-Fe_2_O_3_/Fe_3_O_4_ heterogeneous nanoparticles is displayed in Figure 1b; obviously, the magnetic α-Fe_2_O_3_/Fe_3_O_4_ heterogeneous nanoparticles had a polycrystalline structure, and the average particle size was approximately 42 nm, which agreed with the result of SEM morphology.

**Figure 2:**
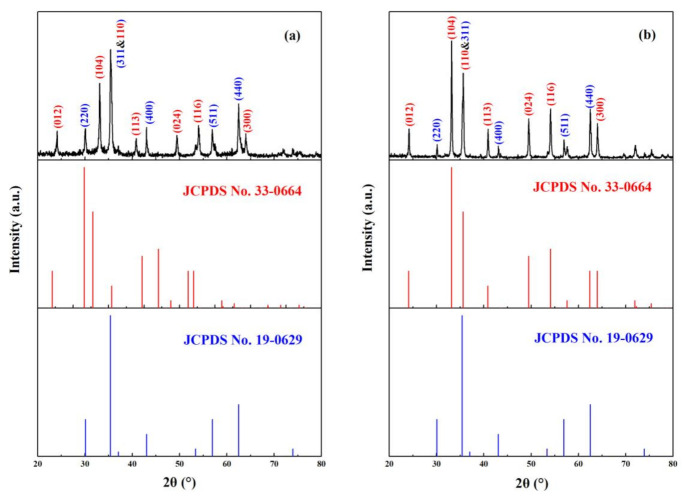
Comparisons of the XRD patterns of Fe_2_O_3_/Fe_3_O_4_ heterogeneous nanoparticles calcined at 400 (**a**) and 500 °C (**b**) for 2 h with the heating rate of 3 °C/min and the molar ratio of ferric nitrate and urea of 1:4 with the standard PDF cards (Fe_2_O_3_: JCPDS No. 33-0664; Fe_3_O_4_: JCPDS No. 19-0629).

Figure 2a,b displays the X-ray diffraction patterns of magnetic α-Fe_2_O_3_/Fe_3_O_4_ heterogeneous nanoparticles calcined at 400 and 500 °C for 2 h with the heating rate of 3 °C/min and the molar ratio of ferric nitrate and urea of 1:4, respectively. Among them, the diffraction peaks of Figure 2a,b basically corresponded to the standard PDF cards of α-Fe_2_O_3_ and Fe_3_O_4_. Compared with the α-Fe_2_O_3_ standard PDF card (JCPDS No. 33-0664) and the Fe_3_O_4_ standard PDF card (JCPDS No. 19-0629), the ratio of diffraction peaks at 33° and 35.6° in Figure 2a were significantly smaller than those in the α-Fe_2_O_3_ standard PDF card, indicating that there was Fe_3_O_4_ diffraction peak at 35.6°. The results proved the existence of Fe_3_O_4_. Although the peak of α-Fe_2_O_3_ also existed at 35.6°, the d-spacing values of Sample A at the diffraction peak at 35.6° (shown in Table 1) belonged to Fe_3_O_4_ instead of α-Fe_2_O_3_ because the d-spacing values (2.5322) matched Fe_3_O_4_ (2.5320) more. In iron oxides, both Fe_3_O_4_ and γ-Fe_2_O_3_ had the same cubic spinel crystalline structure, and it was very difficult to differentiate them with XRD patterns. However, they could be distinguished using d-spacing values. The d-spacing values of Samples A and B, together with the JCPDS files of Fe_3_O_4_ and γ-Fe_2_O_3_, are listed in Table 1. It can be seen that experimental data matched with the d-spacing values of Fe_3_O_4_ better, indicating the formation of Fe_3_O_4_. There are no γ-Fe_2_O_3_-specific diffraction peaks at 26° in the two figures, implying that the other substance was Fe_3_O_4_ instead of γ-Fe_2_O_3_. The difference between Samples A and B was that as the calcination temperature increased, the intensity of the diffraction peak at 35.6° significantly decreased, indicating that the relative content of Fe_3_O_4_ was lowered at 500 °C, but it could not be said that Fe_3_O_4_ disappeared completely after the calcination temperature of 500 °C. The presence of a weak Fe_3_O_4_ diffraction peak can still be seen in Figure 2b. These characterizations confirmed that magnetic α-Fe_2_O_3_/Fe_3_O_4_ heterogeneous nanoparticles were successfully obtained.

### 3.2. Effects of Experimental Conditions on the Ratio of α-Fe_2_O_3_ and Fe_3_O_4_ in Heterogeneous Nanoparticles

**Figure 3:**
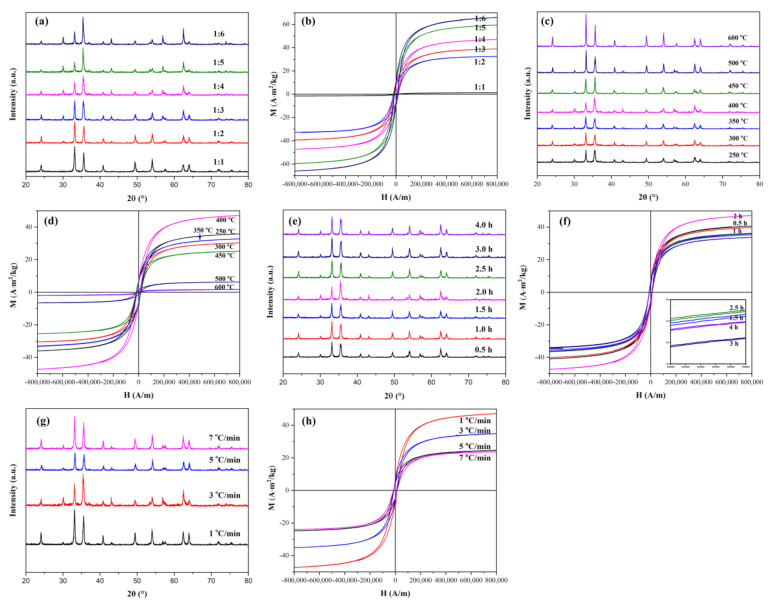
XRD patterns and hysteresis loops of Fe_2_O_3_/Fe_3_O_4_ heterogeneous nanoparticles with various molar ratios of ferric nitrate and urea (**a**,**b**) calcined at various temperatures (**c**,**d**) for different times (**e**,**f**) with different heating rates (**g**,**h**).

The effects of the calcination temperature, the calcination time, the ratio of ferric nitrate and urea, and the heating rate on the relative content of Fe_3_O_4_ in the heterogeneous nanoparticles were investigated by changing the preparation conditions. As mentioned above, the phase composition and grain characteristics of the sample could be analyzed from XRD. However, for this nanomaterial, the magnetic properties of nanoparticles were also needed to be measured in order to better apply them. VSM was a magnetic parameter measurement system that made use of the small sample to make tiny wobbles in the magnetic field and induced the electromotive force from the adjacent coil. The magnetic properties of the heterogeneous nanoparticles could be obtained by comparing the magnetic hysteresis loops measured by VSM. Figure 3a,b shows the XRD and VSM patterns of the heterogeneous nanoparticles obtained at a calcination temperature of 400 °C for 2 h with the heating rate of 3 °C·min^−1^ with various molar ratios of ferric nitrate and urea. As can be seen from Figure 3b, the saturation magnetization of the heterogeneous nanoparticles became larger with the increase of urea amount, and the saturation magnetizations could reach 66.6 A·m^2^/kg when the molar ratio of ferric nitrate and urea was 1:6. Because α-Fe_2_O_3_ showed weak magnetic properties [29], the enhancement of the magnetic properties proved the increase of the Fe_3_O_4_ content in the nanomaterial.

The formation mechanism of Fe_2_O_3_/Fe_3_O_4_ heterogeneous nanoparticles could be understood as similar to the thermal decomposition reaction of [Fe(CON_2_H_4_)_6_](NO_3_)_3_ [30]. As the temperature rose, the water in the mixed solution evaporated quickly, which caused Fe^3+^ to react with CON_2_H_4_ and finally formed [Fe(CON_2_H_4_)_6_](NO_3_)_3_. The thermal decomposition of [Fe(CON_2_H_4_)_6_](NO_3_)_3_ involved a two-stage decomposition process. Fe(NO_3_)_3_ and CON_2_H_4_ were the main products in the first step. The products of the second stage were likely to depend on the heat treatment atmosphere. As the temperature increased, the evaporation of water in the crucible temporarily created a vacuum environment around the crucible. At the same time, the ammonia gas generated by the decomposition of urea would act to isolate oxygen around the crucible. The released CON_2_H_4_ in the first stage could not escape completely from the crucible by evaporation; hence, it could partially reduce Fe^3+^ to Fe^2+^, leading to the formation of Fe_3_O_4_. Fe_2_O_3_ was generated by redundant Fe(NO_3_)_3_ decomposition and also formed by oxidation of Fe_3_O_4_ during calcination.

(1) $$\mathrm{Fe}[\left({\mathrm{CON}}_{2}H_{4}{)}_{6}\right]{\left({\mathrm{NO}}_{3}\right)}_{3}\to 3\mathrm{Fe}{\left({\mathrm{NO}}_{3}\right)}_{3}+{\mathrm{CON}}_{2}H_{4}$$

(2) $$3\mathrm{Fe}{\left({\mathrm{NO}}_{3}\right)}_{3}+{\;\mathrm{CON}}_{2}H_{4}\to {\;\mathrm{Fe}}_{3}O_{4}+{\mathrm{CO}}_{2}+4\mathrm{NO}+7{\mathrm{NO}}_{2}+O_{2}+\;2H_{2}$$

When the ratio of ferric nitrate and urea was 1:1, the product was completely Fe_2_O_3_. It could be considered that the mixed reaction system formed due to the lack of air blocked by the solution, resulting in the inability to form Fe_3_O_4_, or the amount of Fe_3_O_4_ formed by the reaction was so little that Fe_3_O_4_ was completely oxidized to Fe_2_O_3_. When the ratio reached 1:2, Fe_3_O_4_ started to be generated, which was inferred from the sudden increase in magnetism. According to the reaction mechanism, it could be foreseen that the amount of Fe_3_O_4_ was gradually increased with the increase of the amount of urea, but the complete generation of Fe_3_O_4_ did not occur. This was due to the decomposition of urea itself and the oxidation of Fe_3_O_4_ during calcination.

Figure 3c,d shows XRD and VSM patterns of Fe_2_O_3_/Fe_3_O_4_ heterogeneous nanoparticles obtained at different calcination temperatures under preparation conditions. The molar ratio of ferric nitrate and urea was 1:4, the eating rate was 3 °C·min^−1^, and the calcination time was set as 2 h. From the XRD patterns shown in Figure 3c, it can be observed that the diffraction peak became higher and narrower as the temperature increased, indicating that the crystallinity became higher, and the particle size became larger. The ratio of diffraction peaks at 33° and 35.6° became smaller and smaller until the temperature reached 400 °C and rose again after 400 °C. As displayed in Figure 3d, it is apparent that Fe_3_O_4_ began to form at 250 °C due to the higher magnetic properties of Fe_3_O_4_. The content of Fe_3_O_4_ reached the maximum at 400 °C, which illustrated that the reduction reaction completed at 400 °C and partial Fe_3_O_4_ was gradually transformed to α-Fe_2_O_3_ after 400 °C. Because the amount of urea volatilized in the reaction temperature was difficult to control (urea involved in the reaction may be less), it showed an increase in the reaction temperature but a decrease in magnetic properties, such as magnetic properties of the heterogeneous nanoparticles at 250, 300, and 350 °C.

The effect of the calcination time on Fe_2_O_3_/Fe_3_O_4_ heterogeneous nanoparticles was investigated and is shown in Figure 3e,f. The molar ratio of ferric nitrate and urea was 1:4, the heating rate was 3 °C·min^−1^, and the calcination temperature was 400 °C. The saturation magnetization of Fe_2_O_3_/Fe_3_O_4_ heterogeneous nanoparticles reached maximum after 2 h of calcination. Combined with the effect of the calcination temperature on Fe_2_O_3_/Fe_3_O_4_ heterogeneous nanoparticles, the reaction was completed at 400 °C for 2 h. Figure 3g,h shows XRD and VSM patterns of Fe_2_O_3_/Fe_3_O_4_ heterogeneous nanoparticles obtained with different heating rates, the molar ratio of ferric nitrate and urea of 1:4 at the calcination temperature of 400 °C, for a calcination time of 2 h. The difference in magnetic properties caused by various heating rates might be related to the total reaction time (including heating, calcination, and cooling time). In short, 3 °C·min^−1^ became the condition that Fe_3_O_4_ was most generated when it was calcined at 400 °C for 2 h.

### 3.3. In Vitro Cytotoxicity Assay

Increasing the use of metal oxide nanoparticles requires a better understanding of their potential impact on human health. Whether the Fe_2_O_3_/Fe_3_O_4_ heterogeneous nanoparticles can enter the cell also depended on the particle size of the heterogeneous nanoparticles [31,32,33]. In the experiments, Prussian blue staining was first applied to determine whether the nanoparticles entered the cells, and most of the nanoparticles entered the cells through the endocytic pathway. The cytotoxicity of Fe_2_O_3_/Fe_3_O_4_ heterogeneous nanoparticles to human hepatocytes L-02 was further evaluated by the different cytotoxic effects through CCK 8 assay, reactive oxygen species (ROS), lactate dehydrogenase (LDH), MDA, and T-AOC to assess the end points of toxicity.

### 3.4. Prussian Blue Staining

**Figure 4:**
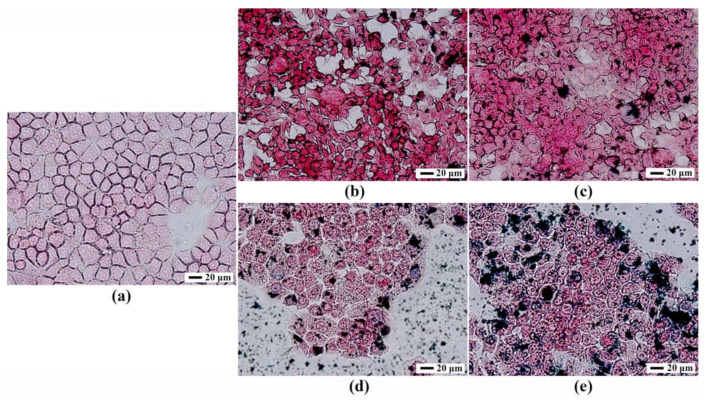
Prussian blue staining of human hepatocytes L-02 incubated by Fe_2_O_3_/Fe_3_O_4_ heterogeneous nanoparticles at 0 (**a**), 50 (**b**), 100 (**c**), 200 (**d**), and 400 ng/mL (**e**).

Prussian blue was a common and sensitive stain to detect the presence of iron in cells or biopsy specimens by pathologists [34]. Ferric iron deposited in tissues and cells reacted with soluble ferrocyanide in the stain and transformed into insoluble Prussian blue dye, a complex hydrated ferric ferrocyanide substance. This allowed Prussian blue to be used to detect whether the material entered into the cells. Figure 4 shows the results of Prussian blue staining. After the Fe_2_O_3_/Fe_3_O_4_ heterogeneous nanoparticles were cocultured with the hepatocytes for 24 h, the Prussian blue staining results visually showed the distribution of the heterogeneous nanoparticles in the cells. Under the inverted microscope, blue iron particles were observed in the cells, indicating that the Fe_2_O_3_/Fe_3_O_4_ heterogeneous entered into the cells. However, the localization to the cytoplasm or nucleus required subsequent electron microscopy.

### 3.5. Cell Counting Kit-8 and LDH Detection of Cell Viability

**Figure 5:**
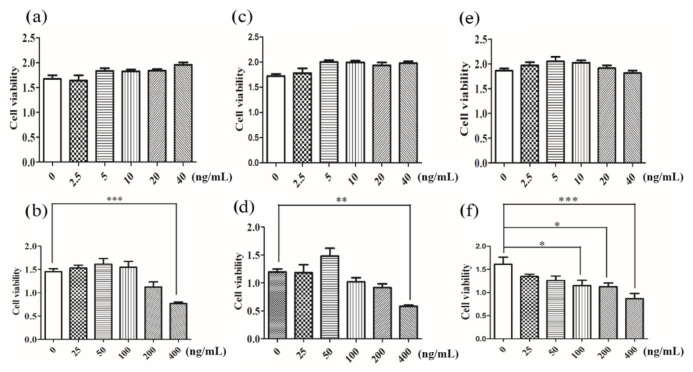
Cell viability assay of Fe_2_O_3_/Fe_3_O_4_ heterogeneous nanoparticles calcined at various temperatures: (**a**,**b**) 250, (**c**,**d**) 300, and (**e**,**f**) 350 °C. * *p* ≤ 0.05, ** *p* ≤ 0.01, *** *p* ≤ 0.001 versus control group, *n* = 6.

Assessment of cellular toxicity was an essential prerequisite to the as-prepared magnetic Fe_2_O_3_/Fe_3_O_4_ heterogeneous nanoparticles in biomedical applications. CCK8 (Cell Counting Kit-8) cell viability assay was a common method to measure the cytotoxicity of drugs [35,36]. Its advantages were accurate, rapid, and stable, and the reagent itself was less cytotoxic. The amount of formazan was proportional to the number of living cells. Therefore, this property could be used to directly perform cell proliferation and toxicity analysis. As evidenced in Figure 5, the results of cell viability demonstrated that the effect of the low dose (0–40 ng/mL) of Fe_2_O_3_/Fe_3_O_4_ heterogeneous nanoparticles on cell viability was not statistically significant, which identified that the Fe_2_O_3_/Fe_3_O_4_ heterogeneous nanoparticles had excellent biocompatibility in the in vitro test. At a high dose (40–400 ng/mL), when the concentration was greater than 100 ng/mL, the cell activities were significantly decreased, which was attributed to the cell biological effects induced by nanoparticles due to the size, morphology, surface charge, composition of the different nanomaterial particles, and the selected cell model. The CCK8 results initially showed that the concentration of Fe_2_O_3_/Fe_3_O_4_ heterogeneous nanoparticles had less effect on cell viability when the concentration was less than 100 ng/mL.

### 3.6. Flow Cytometry Detection of Apoptosis

**Figure 6:**
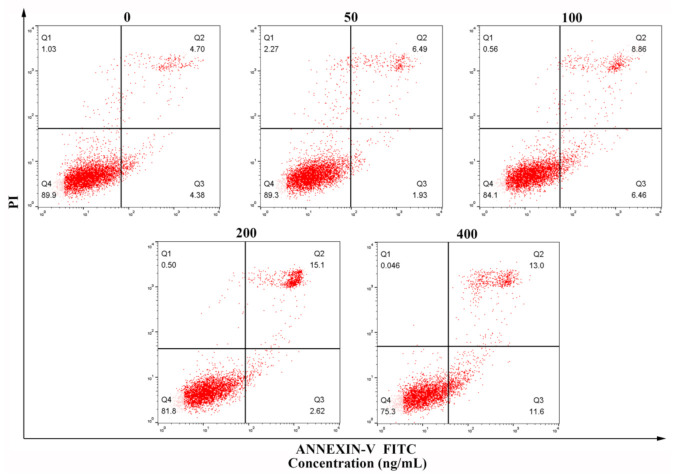
Flow cytometry detection of apoptosis.

Apoptosis might be one of the mechanisms of Fe_2_O_3_/Fe_3_O_4_ heterogeneous nanoparticles cytotoxicity [37]. Apoptosis was the autonomous and orderly death of cells under gene control, which was a process of nuclear condensation, chromosomal DNA degradation, cell shrinkage, and finally the formation of apoptotic bodies [38]. Figure 6 shows that when the concentration of Fe_2_O_3_/Fe_3_O_4_ heterogeneous nanoparticles was more than 100 ng/mL, the apoptosis increased significantly. When the concentration was less than 100 ng/mL, there was no effect on apoptosis.

### 3.7. Elisa Detects MDA Production and T-AOC

**Figure 7:**
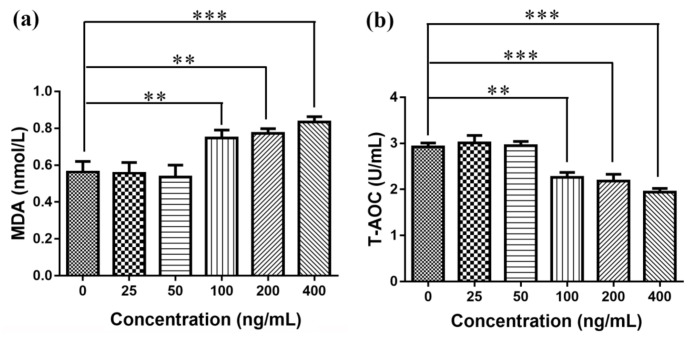
Elisa detection of malondialdehyde (MDA) production and total antioxidant capacity (T-AOC): (**a**) MDA generation and (**b**) T-AOC detection. ** *p* ≤ 0.01, *** *p* ≤ 0.001 versus control group, *n* = 6.

At present, the toxicity mechanism of nanomaterials was mainly oxidative stress, and oxidative stress was one of the basic ways of cell damage in organisms. Oxidative stress referred to the process of oxidative damage caused by the increase or elimination of oxygen free radicals in the body tissues or cells, resulting in the accumulation of reactive oxygen species in the body or cells [39]. ROS could attack biofilm unsaturated fatty acids to form lipid peroxides, and its product MDA was a harmful substance, which could be often used as an indicator to evaluate the strength of lipid peroxidation [40]. T-AOC was the reaction tissue cell enzymatic and an important indicator of the total antioxidant capacity of nonenzymatic systems [41]. This study found that when the concentration was greater than 100 ng/mL, compared with the normal control group, the amount of MDA production in each group was significantly increased, and the T-AOC ability was significantly decreased, as shown in Figure 7. When the concentration was less than 100 ng/mL, compared with the normal control group, the amount of MDA produced and T-AOC ability were not statistically significant.

### 3.8. Blood Routine and Biochemical Assay of Mice

**Figure 8:**
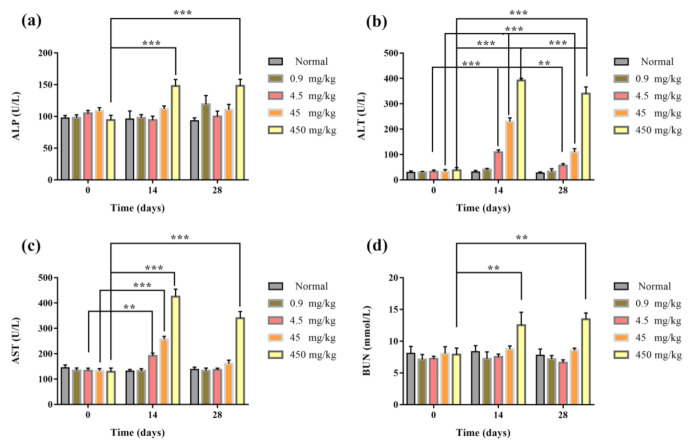
Blood biochemical indexes of mice injected via tail vein with 200 μL of Fe_2_O_3_/Fe_3_O_4_ heterogeneous nanoparticles solutions of various concentrations: (**a**) alkaline phosphatase (ALP), (**b**) alanine aminotransferase (ALT), (**c**) aspartate aminotransferase (AST), and (**d**) blood urea nitrogen (BUN) (the control groups were injected with the same volumes of normal saline). ** *p* ≤ 0.01, *** *p* ≤ 0.001 versus control group, *n* = 6.

**Figure 9:**
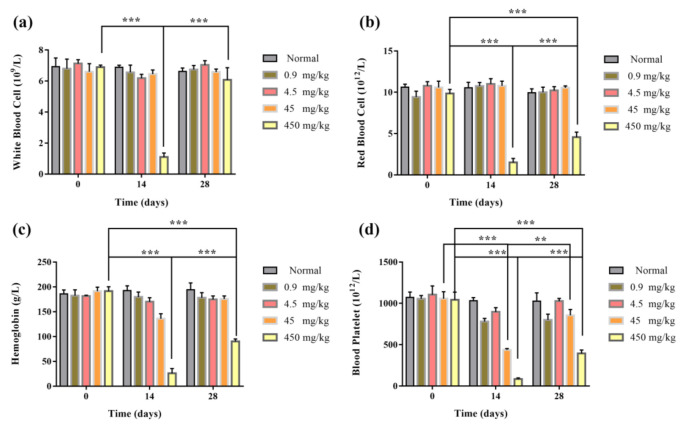
The blood routine indexes of mice injected via tail vein with 200 μL of Fe_2_O_3_/Fe_3_O_4_ heterogeneous nanoparticles solutions of various concentrations: (**a**) white blood cell (WBC), (**b**) red blood cell (RBC), (**c**) hemoglobin (HB), and (**d**) blood platelet (blood) (the control groups were injected with the same volumes of normal saline). ** *p* ≤ 0.01, *** *p* ≤ 0.001 versus control group, *n* = 6.

After the tail vein injection of Fe_2_O_3_/Fe_3_O_4_ heterogeneous nanoparticles, the blood routine and blood biochemical results of mice in each group were detected at Days 0, 14, and 28. The blood routine indexes included white blood cell (WBC), red blood cell (RBC), hemoglobin (HB), and blood platelet (blood). Blood biochemical indexes included alanine aminotransferase (ALT), aspartate aminotransferase (AST), alkaline phosphatase (ALP), and blood urea nitrogen (BUN). The results of the blood routine test showed that the blood routine values of 0.9 and 4.5 mg/kg groups were in the normal range. After 14 days of administration, compared with the control group, the platelet content of the 45 mg/kg group was significantly decreased, and the number of white blood cells in the 450 mg/kg group decreased significantly after 14 days and returned to normal value at 28 days. It might be that the nanomaterials had a certain damage effect on white blood cells (Figure 8). Blood biochemical results showed that the values of ALT and AST in the 4.5, 45, and 450 mg/kg groups were significantly lower than those in the control group after 14 days compared with the control group, indicating that liver injury might have been caused. In the 450 mg/kg group, after 14 days of administration, the renal function index blood urea nitrogen increased significantly, indicating that renal injury might have been caused (Figure 9). In conclusion, the results of the blood routine test and blood biochemistry in the 0.9 mg/kg group were within the normal range within 0–28 days after administration, and the tail vein administration concentration of 0.9 mg/kg had good biological safety.

### 3.9. Histopathological Section Observation of Mice

**Figure 10:**
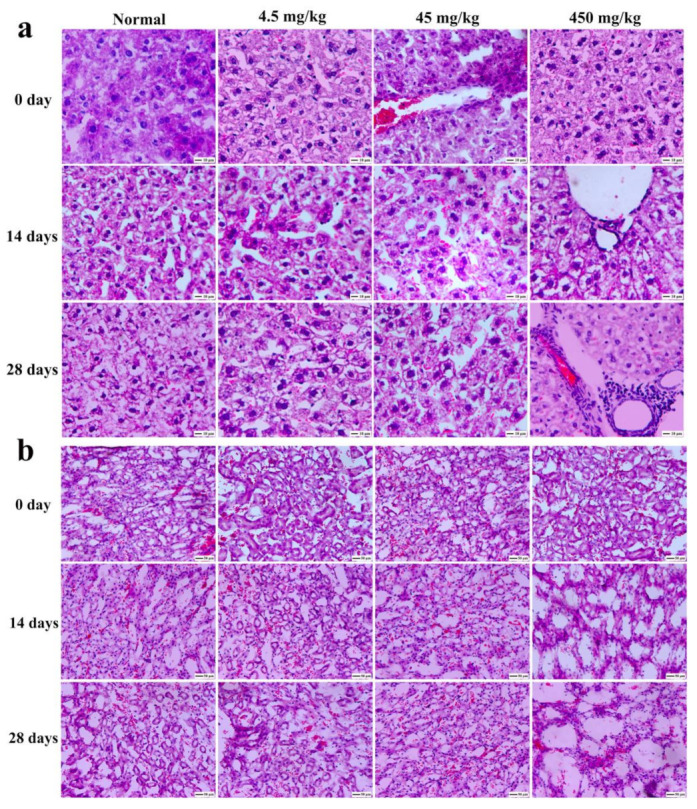
Physiological section of lung from mice of the Fe_2_O_3_/Fe_3_O_4_ heterogeneous nanoparticles (**a**) and control groups (**b**) with different doses (hematoxylin staining).

Figure 10 showed the hematoxylin and eosin (H&E) staining of tumors on various days with different doses of Fe_2_O_3_/Fe_3_O_4_ heterogeneous nanoparticles. It could be observed that the tissue section had no obvious pathological changes within the injection range of 45 mg/kg, and there were no significant differences between a and b, which indicated that the Fe_2_O_3_/Fe_3_O_4_ heterogeneous nanoparticles had high biocompatibility in vivo. Additionally, H&E staining with the injection dose of 45 mg/kg revealed higher apoptosis compared with the control group, confirming that the Fe_2_O_3_/Fe_3_O_4_ heterogeneous nanoparticles induced cell apoptosis at a late stage at a high dose.

## 4. Conclusions

A novel type of magnetic Fe_2_O_3_/Fe_3_O_4_ heterogeneous nanoparticles, which were spherical nanomaterials with a uniform size distribution, was successfully prepared via a facile solution combustion process, using ferric nitrate and urea as raw materials. The average particle size of the heterogeneous nanoparticles was 42 nm, and the content of Fe_3_O_4_ in Fe_2_O_3_/Fe_3_O_4_ heterogeneous nanoparticles was mainly affected by the ratio of ferric nitrate and urea, the reaction temperature, the reaction time, and the heating rate. Under the condition that the ratio of ferric nitrate and urea was 1:4, the Fe_2_O_3_/Fe_3_O_4_ heterogeneous nanoparticles with the highest Fe_3_O_4_ content could be obtained at 400 °C for 2 h with the heating rate of 3 °C·min^−1^. The saturation magnetization strength of the heterogeneous nanoparticles was 47.1 A·m^2^/kg. As the amount of urea increased, the amount of Fe_3_O_4_ naturally increased, and the selectivity was high. The Fe_2_O_3_/Fe_3_O_4_ heterogeneous nanoparticles could enter cells by endocytosis. The CCK8 results initially showed that Fe_2_O_3_/Fe_3_O_4_ heterogeneous nanoparticles had no effect on cell viability when the concentration of nanoparticles was less than 100 ng·mL^−1^. When the concentration was greater than 100 ng·mL^−1^, oxidative stress and apoptosis were observed in L-02 cells, and the heterogeneous nanoparticles displayed cytotoxicity. MDA production increased and the total antioxidant capacity declined. The nanoparticles were cytotoxic, the toxicity was concentration dependent, and they had good biological safety within the concentration of 0.9 mg/kg.

## Figures and Tables

**Table 1:** D-spacings of Samples A and B and those of Fe_3_O_4_, γ-Fe_2_O_3_, and α-Fe_2_O_3_ from the JCPDS files: ^1^ JCPDS file No. 19-0629; ^2^ JCPDS file No. 39-1346; ^3^ JCPDS file No. 33-0664.

Sample A (Å)	Sample B (Å)	Fe_3_O_4_ (Å) ^1^	γ-Fe_2_O_3_ (Å) ^2^	hkl	α-Fe_2_O_3_(Å) ^3^	hkl
2.9666	2.9627	2.9670	2.9530	220	-	-
2.5322	2.5156	2.5320	2.5177	311	2.5190	110
2.1008	2.0980	2.0993	2.0886	400	-	-
1.6159	1.6144	1.6158	1.6073	511	-	-
1.4844	1.4844	1.4845	1.4758	440	-	-

## Data Availability

The data presented in this study are openly available in ICDD-PDF.
